# Ingredient-Dependent Extent of Lipid Oxidation in Margarine

**DOI:** 10.3390/antiox10010105

**Published:** 2021-01-13

**Authors:** Sarah Fruehwirth, Sandra Egger, Dennis Kurzbach, Jakob Windisch, Franz Jirsa, Thomas Flecker, Miriam Ressler, Agnes T. Reiner, Nesrin Firat, Marc Pignitter

**Affiliations:** 1 Department of Physiological Chemistry, Faculty of Chemistry, University of Vienna, 1090 Vienna, Austria; sarah.f.fruehwirth@univie.ac.at (S.F.); Sandra.Egger@senna.at (S.E.); agnes.reiner@univie.ac.at (A.T.R.); 2 Senna Nahrungsmittel GmbH & Co KG, 1140 Wien, Austria; Nesrin.Firat@senna.at; 3 Institute of Biological Chemistry, Faculty of Chemistry, University of Vienna, 1090 Vienna, Austria; dennis.kurzbach@univie.ac.at; 4 Institute of Inorganic Chemistry, Faculty of Chemistry, University of Vienna, 1090 Vienna, Austria; jakob.windisch@univie.ac.at (J.W.); franz.jirsa@univie.ac.at (F.J.); 5 Department of Zoology, University of Johannesburg, Auckland Park, Johannesburg 2006, South Africa; 6 Josef Ressel Center for Development of Comprehensive Analytical Tools for the Pharmaceutical Industry, Department of Biomedical Science, FH JOANNEUM, 8020 Graz, Austria; flecker.th@gmail.com (T.F.); Miriam.Ressler@fh-joanneum.at (M.R.)

**Keywords:** margarine, lipid oxidation, W/O emulsions, food additives

## Abstract

This study reports the impact of margarine-representative ingredients on its oxidative stability and green tea extract as a promising antioxidant in margarine. Oil-in-water emulsions received much attention regarding factors that influence their oxidative stability, however, water-in-oil emulsions have only been scarcely investigated. Margarine, a widely consumed water-in-oil emulsion, consists of 80–90% fat and is thermally treated when used for baking. As different types of margarine contain varying additives, their impact on the oxidative stability of margarine during processing is of pressing importance. Thus, the influence of different ingredients, such as emulsifiers, antioxidants, citric acid, β-carotene and NaCl on the oxidative stability of margarine, heated at 80 °C for 1 h to accelerate lipid oxidation, was analyzed by the peroxide value and oxidation induction time. We found that monoglycerides influenced lipid oxidation depending on their fatty acyl chain. α-Tocopheryl acetate promoted lipid oxidation, while rosemary and green tea extract led to the opposite. Whereas green tea extract alone showed the most prominent antioxidant effect, combinations of green tea extract with citric acid, β-carotene or NaCl increased lipid oxidation in margarine. Complementary, NMR data suggested that polyphenols in green tea extracts might decrease lipid mobility at the surface of the water droplets, which might lead to chelating of transition metals at the interface and decreasing lipid oxidation.

## 1. Introduction

Margarine is an emulsion of water droplets in oil (W/O) [1] and used as a low-fat alternative for butter and other fat-soluble spreads [2]. Whereas oil in water emulsions (O/W) have been investigated comprehensively [3,4,5,6,7], W/O emulsions have been studied scarcely and the characterization of their long-term stabilization still remains a challenging task in food science, limiting their potential application in the food industry. It is essential to understand the factors that affect the stability of an emulsion to develop high-quality emulsion-based food products, such as spreads, sauces and margarine [8].

Regarding margarine, this still remains a challenging task as various additives, contained in the product, influence the oxidative stability [8], such as emulsifiers, antioxidants, metal ions, NaCl, citric acid or carotenoids [9,10,11,12].

An emulsion can be divided into an oil phase, an aqueous phase and an interfacial region [13], whereby the interfacial region is the contact region between the oil phase and the aqueous phase representing the critical area of the system where lipid oxidation occurs [1,14]. Since emulsions are thermodynamically unstable systems, emulsifiers are used for their stabilization [3].

Antioxidants can decelerate or stop the oxidation of other substances by acting as radical scavengers, singlet oxygen quenchers or through UV absorption [15,16,17,18]. In emulsions, the polarity [19,20] and the chain length [21] of the antioxidants are of great importance. While hydrophilic, polar antioxidants, such as green tea extract, exhibit stronger impact on the oil phase and are more effective in W/O emulsions, lipophilic, nonpolar antioxidants, such as tocopherols or ascorbyl palmitate, have higher impact on aqueous phases and are more active in O/W emulsions [19,20]. Laguerre et al. [21] investigated the relationship between antioxidant property and hydrophobicity with a complete homologous series of chlorogenic acid esters and observed that the antioxidant capacity increased with increasing alkyl chain length until reaching the length of the dodecyl chain. Further extension of the chain led to a drastic decrease of the antioxidative efficiency [21], known as cutoff effect [22]. Furthermore, the location of the antioxidants strongly influenced their antioxidant properties since it was shown from partition analysis that the concentration of the dodecyl ester in the aqueous phase was the lowest compared to the esters with shorter alkyl chains [21].

Minor components, such as metal ions, NaCl, citric acid and β-carotene have been analyzed in different emulsion systems [23,24,25,26,27], but their impact on the oxidative stability of margarine remains unknown. Dridi et al. [23] analyzed the influence of iron on the lipid oxidation rate in the aqueous phase of a W/O emulsion since iron is one of the major pro-oxidants existing in foods [28]. They could show that the rate of lipid oxidation increased with higher amounts of iron [23].

However, the stability of W/O emulsions could be improved by adding inorganic salts, such as CaCl_2_, NaCl, MgCl_2_ and MgSO_4_, in the aqueous phase [8]. They reduced Ostwald ripening [24], improved the stability against coalescence and sedimentation due to a decreased particle size. Additionally, they reduced the interfacial tension between oil and water phases [26], and lowered the Van der Waals attraction between emulsion droplets [27]. On the contrary, Osborn-Barnes et al. [29] and Mei et al. [30] observed prooxidative effects of inorganic salts in O/W emulsions.

Moreover, citric acid has been shown to influence the physical stability of sunflower O/W emulsions stabilized by 7% (*w*/*w*) bambara groundnut flour. Adeyi et al. [11] controlled and manipulated these emulsions with citric acid as it was shown to increase the oil droplet size relative to emulsions without citric acid. Further, citric acid was able to weaken the bambara groundnut flour matrix by hydrolyzation and changing its molecular conformation, thereby reducing the matrix strength necessary for the formation of an emulsion [11].

β-Carotene, one of the most important fat-soluble pigments [12], is usually added to margarine to imitate the natural color of butter [31]. It is a biological antioxidant that protects cells and tissues from the damaging effects of free radicals [32] and is a potential anti-carcinogen [33]. However, it could be shown that most of the β-carotene, which was added to rapeseed and sunflower oils, oxidized in the first five hours of heating at 110 °C in a rancimat apparatus leading to significant effects on the peroxide index, free fatty acid values and radical scavenging activity [12] suggesting a prooxidant behavior of β-carotene or its oxidized species [34].

The aim of this work was to analyze the influence of different ingredients on the oxidative stability of margarine. Margarine was produced in a pilot facility and the influence of two different emulsifiers, three different antioxidants, i.e., dl-α-tocopheryl acetate, rosemary extract and green tea extract in three different concentrations each as well as minor components, such as citric acid, β-carotene and NaCl were investigated by analyzing the peroxide value and oxidation induction time. Furthermore, the pH-value, iron and copper concentration of the different margarine samples were determined. Finally, nuclear magnetic resonance analysis revealed the impact of the different ingredients on the interfacial region of the W/O emulsion.

We hereby report the impact of different margarine-representative ingredients on its oxidative stability, showing green tea extract as a promising antioxidant in margarine.

## 2. Materials and Methods

### 2.1. Chemicals and Materials

All chemicals were purchased from Merck (Vienna, Austria), Carl Roth (Karlsruhe, Germany) and VWR International GmbH (Vienna, Austria). Solvents used for chromatography were LC‑MS grade.

The margarine consisted of 57% refined palm oil, 12% refined palm stearin, 11% refined rapeseed oil and 20% water. Two different emulsifiers were examined. Emulsifier 1 (E1) consisted of a distilled monoglyceride from hydrogenated palm oil. Emulsifier 2 (E2) consisted of a distilled monoglyceride from non-hydrogenated sunflower oil. The antioxidants DL‑α‑tocopheryl acetate (aTA; Vitamin E Acetat PhE, HAUPT Chemicals GmbH, Wien, Austria), rosemary extract E392 (RE; Winox Fresh, FRUTAROM Savory Solutions Austria GmbH, Salzburg, Austria) and green tea extract (GTE; GUARDIANTM Green Tea Extract 20S, DuPont Danisco, Itasca, IL, USA) were used. The GTE contained 20% catechins.

Citric acid (E330), β-carotene and NaCl were used as minor components.

### 2.2. Production of Margarine

**Figure 1:**
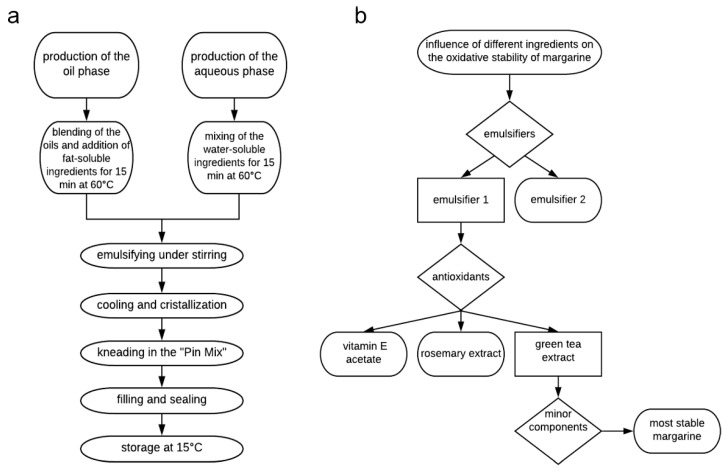
Margarine production scheme (**a**) and study design (**b**).

Margarines were produced at a pilot facility (SCADA SPX Flow Technology, Gerstenberg Schröder), provided by Senna GmbH & Co KG (Vienna, Austria). The pilot facility consisted of an emulsion tank, a pump, two crystallization units and a “Pin Mix”. The production scheme is given in Figure 1a. The fats and oils were mixed in the emulsion tank at 60 °C for 15 min and the fat-soluble ingredients (E1, E2, aTA, RE and β-carotene) were added and dissolved in the oil phase. The water-soluble ingredients (GTE, citric acid and NaCl) were dissolved in the aqueous phase at 60 °C and stirred for 15 min. The two phases were slowly mixed under stirring. A stable W/O emulsion was reached after 30 min of stirring. After cooling and crystallization, the emulsion kneaded in the “Pin Mix”. The pump had a capacity of 80 L/h, the crystallization unit 1 operated at −6 °C and 500 rpm, the crystallization unit 2 at −4 °C and 800 rpm and the rotor of the “Pin Mix” worked at 1000 rpm. The margarine was filled in 0.5 kg packages and sealed with aluminum foil. The margarines were stored for one day at 15 °C for complete hardening. For sampling, the foil was removed and the upper layer of the margarine with a thickness of 1 cm was discarded. Then, samples were aliquoted by taking 50 g of margarine. Samples were directly weighed in amber glass vials (20 mL) after cleaning of the glass vials to remove all traces of lipids and lipid oxidation-promoting agents [35]. For cleaning, the amber glass vials were soaked for 1 h with ethanol saturated with sodium hydroxide. Then, they were rinsed three times with double-distilled water and filled for 1 h with 1 N HCl. Finally, the vials were rinsed three times with double-distilled water and dried overnight upside down in a compartment dryer [35].

To accelerate lipid oxidation, the amber glass vials, containing 10.0 ± 0.01 g of margarine, were heated in an oven (DRY-Line ^®^, VWR International GmbH, Radnor, PA, USA) for 1 h at 80 °C before analysis. Samples for measuring the oxidative stability with the rancimat method (20 g) were melted for 25 min at 80 °C before analysis. Samples (n = 4) were stored under argon atmosphere at −80 °C till sample preparation.

### 2.3. Study Design

The influence of different ingredients, such as emulsifiers, antioxidants and three minor components, citric acid, β-carotene and NaCl, on the oxidative stability of margarine was analyzed in this study. Therefore, a basic margarine was produced that consisted of 57% refined palm oil, 12% refined palm stearin, 11% refined rapeseed oil and 20% water.

In the first testing step, the influence of two different emulsifiers (E471) in three different concentrations—a commonly applied concentration, a 20% lower and a 20% higher concentration—was tested (Figure 1b). The concentrations of E1 and E2 represent the market-based standard concentrations, which are usually applied in margarines in Europe. Therefore, different concentrations of E1 and E2 were used for comparison in this study, which might be a limitation of this study. The commonly applied concentration was chosen according to market-based concentrations and the Codex Alimentarius.

In the second testing step, the influence of different antioxidants in three different concentrations—a commonly applied concentration, a 20% lower and a 20% higher concentration—was tested on the oxidative stability of the margarine, produced with the commonly applied concentration of E1. Therefore, three antioxidants, DL-α-tocopheryl acetate, rosemary extract and green tea were used.

Finally, the individual influence of citric acid, β-carotene and NaCl on the oxidative stability of margarine was tested by using the margarine with the commonly applied concentration of E1 and GTE at the 20% lower concentration. Additionally, a margarine was produced that contained all minor components.

All ingredients are listed in their respective concentrations in Table 1.

### 2.4. Analysis of the Fatty Acid Composition by GC/FID

Fatty acids from margarine were analyzed as their respective fatty acid methyl esters (FAMEs) via GC/FID (Agilent 7890A, Vienna, Austria). Sample preparation and GC/FID analysis were performed in line with the method C-VI 10a from the German Society for Fat Science (DGF) [36] and according to Fruehwirth et al. [37]. In brief, 50 g of margarines were heated at 80 °C in an oven (DRY-Line^®^, VWR International GmbH) until phase separation and 200 µL of the fat phase was combined with 2 mL of isooctane in a test tube. Subsequently, 100 µL of a methanolic potassium hydroxide solution (1.2 M) were added and the tube was shaken for 30 s. A drop of a 0.1% methyl orange solution was added as indicator. Then, the pH was adjusted with 1 N HCl until it reached a value less than 4.0 and the upper phase was used for analysis. Analyses of four independent replicates were performed.

A HP-88 (88% cyanopropyl)aryl polysiloxane column (100 m × 250 µm × 0.2 µm, Agilent Technologies Österreich GmbH, Vienna, Austria.) was used for performing the GC/FID analysis with the following temperature gradient in 20 min: 100 °C for 4 min, then increase to 170 °C within 6 min and to 240 °C within 10 min. Samples were injected (2 µL) with a split ratio of 5:1 and a split flow of 10 mL/min at 250 °C. Carrier gas consisted of hydrogen with a column flow of 2 mL/min, whereas gases for the FID were nitrogen (25 mL/min), hydrogen (30 mL/min), and synthetic air (300 mL/min).

The percental distribution of the fatty acids was evaluated by comparing the area of each FAME with the total area of all FAMEs, which was used for quantitation. Retention times were determined by the use of standards.

### 2.5. Measurement of the Peroxide Value

The peroxide value (PV) was measured according to the method of Wheeler [38] and DGF C-VI 6a [36]. An automatic titration unit (Titrino plus 848, Metrohm Inula GmbH, Vienna, Austria) with electrochemical endpoint determination was used for the analysis. A total of 2.5 g margarine was melted at 50 °C and dissolved in 20 mL acetic acid/isooctane (3:2, *v*/*v*). Then, 200 µL of a saturated potassium iodide solution was added and 80 mL bidestilled water were added under stirring of the sample.

The titrant solution (1 mM Na_2_S_2_O_3_) was added by the automatic titration unit until the equivalence point was reached and the PV, indicated in meq O_2_/kg oil, was calculated automatically.

### 2.6. Detection of the Oxidation Induction Time

The oxidative stability of margarine was determined using the rancimat method by applying the rancimat apparatus (Metrohm 743, Herisau, Switzerland) [39,40]. A total of 20 g of margarine were melted in an oven (DRY-Line^®^, VWR International GmbH) at 80 °C for 25 min. Rancimat vessels containing 3 g of margarine were used for the analysis. The air rate was 10 L/h. The oxidation induction time (OIT, in hours) was evaluated using a temperature of 120 °C. It has to be considered that the aqueous phase of the margarine and the ingredients of the aqueous phase might have an impact on the results of the rancimat test.

### 2.7. NMR Analysis

^1^H R_1_ relaxation rates were obtained on a Magritek SpinSolve Phosphorous spectrometer operating at room temperature and a static magnetic field of B0 = 1 T. The external lock of the spectrometer was used instead of a lock solvent. The inversion recovery method was employed with a recycling delay of 10 s. Spectra were recorded for 20 different recovery delays with a maximum delay time of 4 s. The 90° pulse length was 11.2 µs.

^1^H R_2_ relaxation rates were obtained on the same spectrometer with the same parameters but using the CPMG technique with an echo time of 500 µs. Spectra were recorded for 20 different relaxation delays with a maximum delay time of 1 s.

All data were treated with home-written scripts using the Matlab 2019 software package. All data were zero-filled and apodized using a Gaussian window function prior to Fourier transformation.

As the margarine samples were very inhomogeneous and viscous the linewidths were quite broad, such that only three signals could be discerned, that of water, the bulk of methylene and methyl groups as well as the bulk of CH moieties at the glycerol backbone (Figures S1 and S2). The three signals were deconvoluted by fitting to three Lorentzian lines using the “fitnlorentzian” function for Matlab.

The thus determined signal integrals S(t) were then fitted by mono-exponential functions.

For $R_{1}$ to

(1) $$S\left(t\right)=S_{0}(1-\exp\left(-R_{1}t\right))$$

and for $R_{2}$ to

(2) $$S\left(t\right)=S_{0}\exp\left(-R_{2}t\right)$$

where $S_{0}$ indicated the signal intensity at *t* = 0. *t* is the relaxation delay.

### 2.8. Determination of Iron and Copper

Frozen samples were liquified in a water bath at approximately 45 °C and shaken vigorously to guarantee homogeneity. Then, approximately 0.4 g were weighed into teflon tubes in which they were digested in 9 mL of 34% HNO_3_ (Suprapur^®^ Supelco, Saint Louis, MA, USA) and 1 mL of 30% H_2_O_2_ (Suprapur^®^ Supelco) using a microwave MARS XPRESS system (CEM Corporation, Matthews, NC, USA). The digested samples were transferred into 15-mL volumetric flasks and brought up to volume using Milli-Q^®^ water. Reference samples of fish liver (DOLT-5) obtained from the National Research Council Canada (NRCC) were digested and diluted in the same manner as described above. Iron concentrations were then determined using total x-ray reflection fluorescence spectrometry (S2 PicoFox TXRF, Bruker, Billerica, MA, USA) and copper was determined using graphite furnace atomic absorption spectrometry (GFAAS) using a PinAAcle 900Z (Pelkin Elmer, Waltham, MA, USA).

Recovery rates for elemental analyses in reference samples were 95.5% for Fe and 97.3% for Cu respectively, demonstrating the appropriateness of sample preparation and measurements.

### 2.9. Determination of the pH-Value

A pH meter, which was integrated in the automatic titration unit (Titrino plus 848, Metrohm Inula GmbH, Vienna, Austria), was used for the determination of the pH-value and calibrated before each measurement with two different buffer solutions—pH 4.01 and pH 7. A beaker with a volume of 0.25 L was filled with margarine and heated at 80 °C in an oven for two hours until phases were separated. Subsequently, the aqueous phase was pipetted in a 50 mL beaker and cooled until it reached room temperature. A glass electrode was used for the exact determination of the pH-value.

### 2.10. Statistical Analysis

Data were analyzed using Microsoft Excel and SigmaPlot 14.0 (Synstat Software GmbH, Erkrath, Germany). All results of the PV, OIT and pH-value are shown as mean ± SD of four independent experiments (n = 4). NMR results were determined as single measurements. Data of the determination of copper and iron are depicted as mean ± SD of three independent measurements (n = 3). The Nalimov test was used to exclude outliers. Statistical analysis was performed via one-way analysis of variance (ANOVA) and Holm–Sidak post-hoc test. If data did not exhibit normal distribution, results were determined via Kruskal–Wallis ANOVA followed by Dunn’s post-hoc test.

## 3. Results and Discussion

### 3.1. Fatty Acid Composition of Margarine

The margarine M(total), that contained green tea extract (0.160%), emulsifier 1 (0.175%) and all minor components in their respective concentration according to Table 1, was characterized regarding its fatty acid composition. The fatty acid composition was only characterized for M(total) as the matrix stayed the same for all margarines used in this study. They only differed in their composition regarding antioxidants, emulsifiers and minor components, which should have no effect on the fatty acid composition as it has been proven that the fatty acid composition might not be a reliable and sensitive marker for assessing the lipid oxidation status [41].

The main constituents were oleic acid with 41.6 ± 0.3% and palmitic acid with 40.4 ± 0.0%, due to the large amounts of palm oil in the margarine [42]. Further, large amounts of linoleic acid were found with 10.7 ± 0.1%, due to the rapeseed oil in the margarine [43].

### 3.2. Ingredient-Dependent Influences on the Oxidative Stability of Margarine

The influence of different emulsifiers, dl-α-tocopheryl acetate (aTA), rosemary extract (RE), green tea extract (GTE), citric acid, β-carotene and NaCl on the oxidative stability of margarine was analyzed by determining the PV and OIT of the differently produced margarines. The measurement of the PV was used to determine primary oxidation products and the rancimat method to determine secondary oxidation products. It has to be noted that the PV analysis was conducted at room temperature, whereas the rancimat method was carried out under forced thermal conditions.

#### 3.2.1. Influence of Different Emulsifiers

Emulsions, such as margarine, are thermodynamically unstable systems. Therefore, emulsifiers are used for their stabilization [3], by reducing the interfacial tension between the two immiscible phases [10]. Emulsifiers can enhance the stability of emulsions and act as physical barriers between the oil phase and the aqueous phase, thereby preventing oxidant penetrating and diffusion [13].

**Figure 2:**
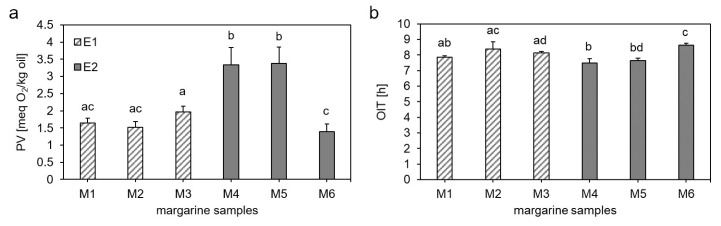
Influence of different emulsifiers on the oxidative stability of margarine determined by the peroxide value (**a**) and oxidation induction time (**b**). M1–M3 consisted of margarines using E1 in commonly applied concentration, 20% lower concentration and 20% higher concentration, respectively. M4–M6 consisted of margarines using E2 in commonly applied concentration, 20% lower concentration and 20% higher concentration, respectively. Data are depicted as mean + SD (n = 4). Statistically significant differences between the margarine samples are indicated with different lower-case letters (one-way ANOVA, Holm–Sidak, *p* < 0.05).

The effects of two different emulsifiers at three different concentrations were investigated by determining the PV and OIT of the differently produced margarines M1–M6 (Table 1, Figure 2). It could be shown that the PVs in margarine prepared with E1 (M1–M3) did not differ between the different concentrations (Figure 2a). The same picture emerged by analyzing the OITs of the respective margarine samples (Figure 2b). However, the PV increased from 1.64 ± 0.14 meq O_2_/kg oil in the margarine M1 to 3.33 ± 0.51 meq O_2_/kg in the margarine M4, which consisted of the commonly applied concentration of E1 and E2, respectively. Using E2 (M4–M6), no differences could be determined between the margarine sample with the commonly applied concentration (M4) and the one containing the 20% lower concentration (M5) of E2, but the PV decreased to 1.97 ± 0.17 meq O_2_/kg oil in the margarine sample with a 20% higher concentration of E2 (M6). The OITs of margarine samples with E2 increased from 7.74 ± 0.30 h and 7.64 ± 0.14 h in M4 and M5, respectively, to 8.64 ± 0.12 h in M6. This increase fitted to the results of the PV as M6 had a lower PV than M4 and M5.

Summarized, the results showed that the oxidative stability of margarines produced with E1 were independent of the different concentrations, whereas margarines produced with E2 showed concentration-dependent results. The latter results could be confirmed by Yi et al. [44] who analyzed water in walnut oil emulsions and showed that the oxidative stability of the emulsions depended on the concentration of the emulsifier. The formation of lipid hydroperoxides and hexanal decreased with increasing concentrations of the emulsifier polyglycerol polyricinoleate (PGPR). They assumed that excess surfactant might solubilize lipid hydroperoxides out of the oil–water interface, which could result in the decreased lipid oxidation rates in W/O emulsions. Further, Dridi et al. [23] analyzed the influence of the emulsifiers distilled monoglycerides (DMG) and PGPR on the oxidative stability of five different W/O emulsions. By changing the PGPR to DMG ratio, they could show that increasing the PGPR fraction slowed down the lipid oxidation process. They hypothesized that the polar moieties in the PGPR chains are larger than in the DMG molecules, thereby building up thick interfacial layers that act as physical barrier that separated lipid substrates from metals, such as iron, in the aqueous phase [23]. In the current study, both emulsifiers were monoglycerides rendering a conclusion to associate their different effects on lipid oxidation with building up physical barriers at the interface unlikely. However, the emulsifiers were isolated from palm oil and sunflower oil, respectively, which differ in their degree of unsaturation. E2 from sunflower oil promoted lipid oxidation compared to E1, suggesting a significant impact of the acyl chain of monoglycerides on lipid oxidation in margarine. Increasing the concentration of E2 from sunflower oil reversed the effect, most likely by removing oxidized lipids from the interface and the formation of mixed micelles [44]. Further, the different concentrations of E1 and E2 might affect the impact of E1 and E2 on the oxidative status of margarine as well.

Thus, E1 at the commonly applied concentration was used as emulsifier for all further margarine samples.

#### 3.2.2. Influence of Different Antioxidants

**Figure 3:**
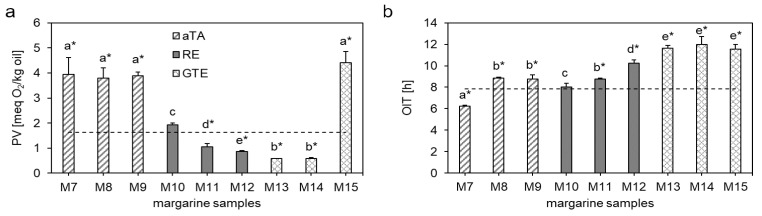
Influence of different antioxidants on the oxidative stability of margarine determined by the peroxide value (**a**) and oxidation induction time (**b**). All margarine samples were produced using E1 in the commonly applied concentration. The influence of DL-α-tocopheryl acetate in commonly applied concentration, 20% lower concentration and 20% higher concentration was analyzed in samples M7–M9, respectively. The influence of rosemary extract in commonly applied concentration, 20% lower concentration and 20% higher concentration was analyzed in samples M10–M12, respectively and the influence of green tea extract in commonly applied concentration, 20% lower concentration and 20% higher concentration was analyzed in samples M13–M15, respectively. Data are depicted as mean + SD (n = 4). Statistically significant differences between the margarine samples are indicated with different lower-case letters (one-way ANOVA, Holm–Sidak, *p* < 0.05). The asterisks show a significant difference to the control. M1 was used as control (dashed line, 1.64 meq O_2_/kg oil (**a**), 7.86 h (**b**)).

Three different antioxidants, aTA, RE and GTE in three different concentrations were applied to produce the different margarines M7-M15 (Table 1). The PVs and OITs of the margarine samples are depicted in Figure 3.

Regarding aTA (M7-M9), it could be analyzed that the PVs increased from 1.64 ± 0.14 meq O_2_/kg oil in M1 to 3.95 ± 0.67 meq O_2_/kg oil in M7, 3.79 ± 0.41 meq O_2_/kg oil in M8 and 3.89 ± 0.14 meq O_2_/kg oil in M9. Thus, aTA acted as a prooxidant in margarine. Martin-Rubio et al. [45] studied the effects of 0.02–2% γ-tocopherol on the accelerated storage-induced lipid oxidation in refined soybean oil by ^1^H NMR and compared it with α‑tocopherol. They could demonstrate that both tocopherol homologues increased the formation of hydroperoxides. Furthermore, in the case of γ-tocopherol, the higher the enrichment degree, the higher the delay in the generation of most secondary oxidation products, which was contrary to α-tocopherol [45].

For the margarine with the commonly applied concentration of aTA (M7) showed the lowest OIT, whereas M8 and M9 showed high OITs with 8.84 ± 0.10 h and 8.75 ± 0.41 h, respectively. The OITs of M8 and M9 were even higher than that of M1 (*p* < 0.05). Kirkhus et al. [46] compared the OITs of olive oil, rapeseed oil, soybean oil, high linoleic sunflower oil, high oleic sunflower oil, and liquid margarine at 90, 120, 160 and 180 °C. Whereas the OIT of liquid margarine at 90 °C was lower than that of olive oil, high linoleic sunflower oil and high oleic sunflower oil, it was higher than all five oils at 160 °C and 180 °C. The authors explained the higher stability of margarine at higher temperatures by a protective effect of water [47,48,49]. Dana et al. [47] assumed that water bubbles, which are injected into heated oil, may act protective by a distillation effect that drives out oxidized volatiles and free radicals formed through deep-frying processes. Kirkhus et al. [46] showed that the water in margarine evaporated within the first 1.5 min of heating and that the measurements of the OIT clearly indicated that volatile compounds were driven off with the water during this period. These results might explain why the margarine samples that contained aTA showed high OIT values and simultaneously exhibiting high PVs.

Regarding the effects of RE (M10-M12), it could be demonstrated that the PVs of sample M1 and sample M10, which consisted of the commonly applied RE concentration, did not differ from each other. However, the PV decreased in the samples that consisted of 20% lower (M11) and higher (M12) concentration of RE with 1.06 ± 0.13 meq O_2_/kg oil and 0.86 ± 0.04 meq O_2_/kg oil, respectively. A change in the concentration of RE led thereby to different PVs. The same effect could be shown by analyzing the OIT. Whereas M10 showed the same results as M1, M11 and M12 showed higher OITs with 8.76 ± 0.09 h and 10.2 ± 0.30 h, respectively. The antioxidative effects of rosemary extract have been proven in different foods [50], such as chicken frankfurters [51] and cooked beef meatballs [52] and are mainly attributed to the phenolic diterpenes, carnosol and carnosic acid [53]. These phenolic diterpenes can act as potent metal chelators and free radical scavengers. Additionally, they effectively inhibited mitochondrial and microsomal lipid peroxidation [54] and showed antimicrobial and antilisterial activity [50].

The lowest PVs were analyzed in samples M13 and M15, which consisted of GTE in the commonly applied and 20% lower concentration, respectively. M13 exhibited a PV of 0.58 ± 0.01 meq O_2_/kg oil and did not differ from M14, which showed a PV of 0.59 ± 0.04 meq O_2_/kg oil. Interestingly, the PV of M15, which contained GTE at a 20% higher concentration, increased to 4.41 ± 0.44 meq O_2_/kg oil. Whereas the PV depended on the different concentrations of GTE, the OIT showed no differences between the three concentrations. All three samples using GTE as antioxidant exhibited the highest OIT compared to the other samples with added antioxidants with 11.6 ± 0.29 h, 12.0 ± 0.72 h and 11.5 ± 0.44 h for M13, M14 and M15, respectively.

Literature shows that GTE can act antioxidative [55,56,57,58] as well as prooxidative [59,60,61]. Chen et al. [55] compared the antioxidative activity of green tea catechin extract and RE. Evaluation of oxygen consumption at 100 ± 2 °C revealed that green tea catechin extract was much more effective against lipid oxidation in canola oil, pork lard and chicken fat than RE [55]. Mustafa [56] showed that GTE inhibited the formation of lipid hydroperoxides in ground beef during refrigerated storage. Senanayake [57] described that GTE was successfully used to extend the shelf-life of various food products and is particularly suitable for products with high susceptibility to lipid oxidation, such as margarine. Despite these findings, Lambert and Elias [59] reported that although catechins are antioxidants, which can chelate transition metals and quench free radical species, there is evidence that some of the effects of these compounds may be related to induction of oxidative stress. The prooxidant activity of green tea polyphenols was also confirmed by Azam et al. [60], who compared the prooxidant properties of epicatechin and epigallocatechin-3-gallate and showed that epigallocatechin-3-gallate was more effective as a prooxidant by reducing Cu(II) to Cu(I), which led to the formation of reactive oxygen species. Moreover, Huang and Frankel [61] showed that tea catechins exhibited different antioxidant activities depending on the lipid system. Whereas epigallocatechin, epigallocatechin gallate and epicatechin gallate were better antioxidants than epicatechin and catechin in oxidized corn oil triglycerides at 140 µM, they all acted prooxidative in the corresponding corn oil in water emulsion at 5 µM and 20 µM by accelerating hydroperoxide and hexanal formation. The authors explained the variation in activity by their different reducing potentials, stabilities and relative distribution between the phases in different lipid systems [61].

Further, green tea extract exhibits high amounts of caffeine [62], which could influence the oxidation status of margarine. Caffeine has been reported as a promising antioxidant in several studies [63,64,65,66,67] by scavenging hydroxyl radicals [63,64,65] as well as superoxide radicals [67].

The results of the different studies might explain why GTE decreased the PV at the commonly applied and 20% lower concentration but increased the PV at the 20% higher concentration.

Therefore, M14, which was produced with E1 in the commonly applied concentration and GTE at a 20% lower concentration was used for the production of further margarine samples.

#### 3.2.3. Influence of Minor Components

The influence of citric acid (M16), β-carotene (M17) and NaCl (M18) on the oxidative stability of margarine was analyzed and compared with margarine containing E1 with (M14) and without GTE (M1).

Citric acid was added to the aqueous phase of the margarine to reach a content of 0.01% citric acid in the margarine. Citric acid is usually added to margarine to decrease the pH-value, which increases the microbial shelf life of the product and contributes to fluffier doughs [31]. The addition of citric acid decreased the pH-value to 3.77 ± 0.00 in M16 (*p* < 0.05), compared to the controls M1 and M14 which showed pH-values of 7.82 ± 0.01 and 4.30 ± 0.00, respectively (Table 2). Table 2 shows that not only citric acid decreased the pH-value of the margarines, but GTE as well. GTE was dissolved in the aqueous phase. Obviously, GTE was acidified by the manufacturer (DuPont Danisco, USA) as the stability of GTE depends on the pH-value [57]. Catechins were shown to exhibit higher stability under acidic conditions (pH < 4) than in basic solutions (pH > 8), where they were converted to their corresponding isomers, which are less stable [57].

**Figure 4:**
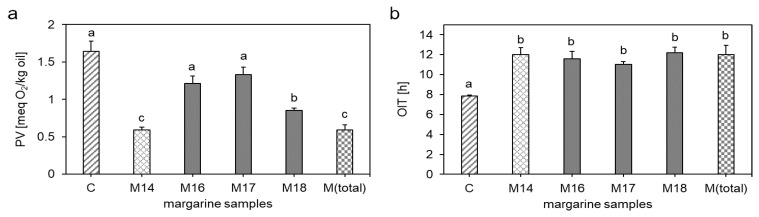
Influence of minor components on the oxidative stability of margarine determined by the peroxide value (**a**) and oxidation induction time (**b**). M1 was used as control (C, 1.64 meq O_2_/kg oil (**a**), 7.86 h (**b**)). M14 represents the margarine without minor components using E1 and 0.160% GTE. Individual components were added to M14. Citric acid was added as minor component in M16, β‑carotene in M17 and NaCl in M18. All minor ingredients were added in M(total). Data are depicted as mean + SD (n = 4). Statistically significant differences between the margarine samples are indicated with different lower-case letters (one-way ANOVA, Holm–Sidak, *p* < 0.05).

Further, the addition of citric acid led to an increase of the PV from 0.59 ± 0.04 meq O_2_/kg oil in M14 to 1.21 ± 0.10 meq O_2_/kg oil in M16 (Figure 4a). Mei et al. [30] showed that a decreased pH-value could increase lipid oxidation as metal ions exhibited a higher solubility at lower pH-values, thereby reaching the oil phase in O/W emulsions more easily. Wang and Wang [68] demonstrated that a decrease of the pH-value from 7.0 to 3.0 increased the attraction between oil droplets and metal ions. Since transition metals are inadvertently introduced into food as part of the ingredients or processes during manufacturing [9] and are therefore inevitable in processed foods, the effect of pH on lipid oxidation via metals has to be considered in the context of margarine stability.

The OIT (Figure 4b) showed no differences between M14 and M16. It can be concluded that the addition of citric acid and the corresponding decrease of the pH-value did not affect the formation of secondary lipid oxidation products, whose volatile degradation products were detected with the rancimat apparatus. A possible explanation for the similar OITs of M14 and M16 despite different PVs could be that prooxidants, such as citric acid, might be partially decomposed at the elevated temperature in the rancimat apparatus [69].

β-Carotene was added together with rapeseed oil as carrier in a concentration of 0.001% to the margarine (M17). β-Carotene is usually used to imitate the natural color of butter [31]. The addition of β-carotene increased the PV from 0.59 ± 0.04 meq O_2_/kg oil in M14 to 1.33 ± 0.10 meq O_2_/kg oil in M17 (Figure 4a). The prooxidative behavior of β-carotene can be referred to the instability of carotenoids. Zeb et al. [12] showed that by adding β-carotene to corn, rapeseed and sunflower oils, most of it oxidized in the first five hours of heating at 110 °C in a rancimat apparatus. Moreover, the addition of β-carotene yielded in significant effects on the peroxide index, free fatty acid values and radical scavenging activity [12], as thermally oxidized β-carotene products can increase the peroxide value of oil, thereby showing a prooxidant behavior of β-carotene or its oxidized species [34]. As the here described margarine was heated at 80 °C for 1 h to accelerate lipid oxidation, it can be assumed that the carotenoids oxidized, which led to an increase of the PV.

Figure 4b shows that the OITs of the margarine samples consisting of the individual minor components (M16–M18) did not differ from M14, which can be explained by the thermal degradation of prooxidants, such as β-carotene, in the rancimat apparatus [70].

NaCl is added to margarine due to its flavoring and antimicrobial effects. In this study, NaCl was added in a concentration of 0.1% in sample M18, which conforms to the average salt content in margarines in Europe [31]. By adding salt to M14, the PV increased significantly to 0.85 ± 0.03 meq O_2_/kg oil in M18, but not as much as by adding citric acid or β-carotene (Figure 4a). Regarding the prooxidative effects of NaCl, literature shows contradictory results. Whereas some studies identified antioxidative effects of NaCl in O/W [71] and W/O emulsions [24,26], others found prooxidative effects in O/W emulsions [29,30]. The prooxidative effect of NaCl can be explained by an increase of the water solubility and catalytic activity of metal ions through chloride ions [72]. Further, monovalent salts, such as NaCl or KCl, were shown to increase the rate of lipid oxidation by affecting the physical properties of the surfactant layer at the oil droplet surface [9].

Summarized, by analyzing the influence of individual components on the oxidative stability of margarine, it could be shown that all three minor components, citric acid, β-carotene and NaCl increased the PV compared to M14, whereas citric acid and β-carotene led to higher PVs than NaCl (*p* < 0.05). No differences could be obtained by analyzing the OITs of the samples M16, M17 and M18.

Further, the influence of all minor components combined was investigated.

#### 3.2.4. Combinational Effects

To evaluate the combinatorial effects of citric acid, β-carotene and NaCl on the oxidative stability of margarine, the margarine sample M(total) was analyzed. M(total) consisted of M14 and contained all minor ingredients in their respective concentrations according to Table 1. Interestingly, a combination of all minor ingredients did not increase the PV, as shown before by analyzing the components individually (Figure 4a). M(total) exhibited a PV of 0.59 ± 0.07 meq O_2_/kg oil as shown for M14, which exhibited a PV of 0.59 ± 0.04 meq O_2_/kg oil. The less pronounced formation of peroxides in M(total) can be explained by the potential of GTE and citric acid to chelate prooxidative transition metals in the aqueous phase [46,59]. The antioxidative effect of citric acid by chelating the transition metals seems to predominate only in the presence of NaCl and β-carotene, as the use of citric acid only, increased the PV of margarine due to the pH-induced impact on metal ions.

The OIT showed similar values as for M14, M16, M17 and M18 with 12.03 ± 0.89 h (Figure 4b).

To get a better understanding of the structural changes at the interface and the metal abundancy in M(total) leading to a decreased PV, margarine samples were analyzed by NMR and iron and copper were determined.

### 3.3. NMR, TXRF and GFAAS Analysis of Margarine Samples

Table 3 shows ratios ^1^H T_1_/T_2_ for the CH_2_ moieties of the fat’s glycerol backbone, Fe (mg/kg) and Cu (mg/kg) values measured by NMR, TXRF and GFAAS, respectively, in selected margarine samples containing a 20% lower concentration of the additives.

Transition metals are inadvertently introduced into food by the ingredients or processes during manufacturing and are therefore inevitable in processed foods [9].

Margarine prepared with GTE in the presence (M(total)) or absence (M14) of all minor ingredients did not reveal any differences with regard to the copper content. Only M14 showed a significant lower concentration of iron compared to the margarine with RE, suggesting that iron was inadvertently introduced together with RE. Thus, the abundancy of total iron and copper might not explain the different vulnerability of the margarine samples towards lipid oxidation.

The ratio T_1_/T_2_, which was calculated by R_2_/R_1_, is proportional to the rotational correlation time (τ_c_) of a moiety under the assumption of spherical dynamics and hence a measure of local mobility [73]. Hence, T_1_/T_2_ can be used to provide information about the potential mobility restraints of the molecules at the lipid droplet surfaces. Tighter molecular packing at the interface and more restricted environments result in increasing τ_c_ [9]. The margarine sample M14 showed the highest T_1_/T_2_ value with 33.2, indicating a lower mobility at the interface. Thus, it might be conceivable that green tea polyphenols might be integrated into the surfactant monolayer, thereby accumulating at the interface and chelating prooxidative transition metals at the site of lipid oxidation. The polyphenols-induced changes of the structural organization at the water droplet surfaces might also be explained by the ability of the chelating polyphenols to pull transition metals away from the interface [9]. Whereas the content of copper did hardly differ between the different margarine samples, M14 showed the lowest amount of iron with 2.22 ± 0.26 mg/kg. The combination of the low amount of iron and a restricted mobility might be the reason for the decreased lipid oxidation in M14.

The margarine sample M(total) showed a T_1_/T_2_ value of 3.58 which indicates a looser molecular packing at the interface than for M14. It can be assumed that GTE hereby chelated metals inside the water droplets as GTE has the potential to form chelate complexes with iron and copper [1,57]. These metals might be removed from the surface of the water droplets through the formation of complexes and consequently might not have the potential to reach the oil phase where they would promote lipid oxidation [1].

M1 and M8 showed the most pronounced mobility at the interface with a T_1_/T_2_ ratio of 2.67 and 1.47, respectively, which might corroborate the enhanced lipid oxidation compared to the margarine fortified with GTE (M14) by a decreased potential of vitamin E, present in M1 and added in M8, to act as an antioxidant at the interface, the site of lipid oxidation.

## 4. Conclusions

The influence of commonly used ingredients in three different concentrations on the oxidative stability of margarine was determined by the PV and OIT. It could be shown that margarine-representative ingredients significantly affected lipid oxidation.

The use of E1, a distilled monoglyceride from hydrogenated palm oil, as emulsifier showed concentration-independent results, whereas margarines produced with E2, a distilled monoglyceride from non-hydrogenated sunflower oil, showed concentration-dependent results. By comparing the antioxidants aTA, RE and GTE it was shown that aTA acted prooxidative, whereas RE with the 20% lower and 20% higher concentration compared to the commonly applied concentration, and GTE with the commonly applied concentration and the 20% lower concentration acted antioxidative. The sample M14, which consisted of 0.160% GTE and E1 at the commonly applied concentration, showed the lowest PV with 0.59 ± 0.04 meq O_2_/kg oil and the highest OIT with 12.0 ± 0.72 h. All minor components, such as citric acid, β-carotene and NaCl, increased the PV compared to M14. Only the margarine, which contained all minor components, showed a low PV like M14 with a PV of 0.59 ± 0.07 meq O_2_/kg. NMR analysis revealed that GTE at low concentration might decrease lipid oxidation by influencing the structural organization of interfacial layers, which might promote chelating metals at the interface.

As shown in this study, food additives might influence lipid oxidation in W/O emulsions and such effects need to be taken into consideration, when optimizing oxidative stability in emulsions.

## Figures and Tables

**Table 1:** Composition of different margarines. Samples M7-M18 consist of the commonly applied concentration of emulsifier 1 with 0.175%.

**Margarine**	**Emulsifiers**	**Content in Margarine (%)**	
**M1**	emulsifier 1	commonly applied concentration	0.175
**M2**	emulsifier 1	−20%	0.140
**M3**	emulsifier 1	+20%	0.210
**M4**	emulsifier 2	commonly applied concentration	0.750
**M5**	emulsifier 2	−20%	0.600
**M6**	emulsifier 2	+20%	0.900
**margarine**	**antioxidants**	**content in margarine (%)**	
**M7**	vitamin E acetate	commonly applied concentration	0.200
**M8**	vitamin E acetate	−20%	0.160
**M9**	vitamin E acetate	+20%	0.240
**M10**	rosemary extract	commonly applied concentration	0.200
**M11**	rosemary extract	−20%	0.160
**M12**	rosemary extract	+20%	0.240
**M13**	green tea extract	commonly applied concentration	0.200
**M14**	green tea extract	−20%	0.160
**M15**	green tea extract	+20%	0.240
**margarine**	**minor components**	**content in margarine (%)**	
**M16**	green tea extract (0.160%) + citric acid		0.010
**M17**	green tea extract (0.160%) + β-carotene		0.001
**M18**	green tea extract (0.160%) + NaCl		0.100
**margarine**	**minor components**	**content in margarine (%)**	
**M(total)**	contains all minor components as well as green tea extract (0.160%) and emulsifier 1 (0.175%)		

**Table 2:** pH-value of the different margarine samples. Data are depicted as mean ± SD (n = 4). Statistically significant differences between the margarine samples and M1 are indicated with an asterisk. Statistically significant differences between the margarine samples and M14 are indicated with two asterisks (one-way ANOVA, Holm–Sidak, *p* < 0.05).

Margarine	Ingredients	pH
**M1**	E1, SC ^1^	7.82 ± 0.01
**M2**	E1, −20%	7.82 ± 0.00
**M3**	E1, +20%	7.91 ± 0.01
**M4**	E2, SC	7.90 ± 0.00
**M5**	E2, −20%	7.86 ± 0.01
**M6**	E2, +20%	7.88 ± 0.01
**M7**	(E1, SC) + (aTA, SC)	7.89 ± 0.01
**M8**	(E1, SC) + (aTA, −20%)	7.90 ± 0.02
**M9**	(E1, SC) + (aTA, +20%)	7.82 ± 0.09
**M10**	(E1, SC) + (RE, SC)	7.91 ± 0.03
**M11**	(E1, SC) + (RE, −20%)	7.91 ± 0.03
**M12**	(E1, SC) + (RE, +20%)	7.92 ± 0.02
**M13**	(E1, SC) + (GTE, SC)	4.31 ± 0.00 *
**M14**	(E1, SC) + (GTE, −20%)	4.30 ± 0.00 *
**M15**	(E1, SC) + (GTE, +20%)	4.12 ± 0.00 *
**M16**	(E1, SC) + (GTE, −20%) + citric acid	3.77 ± 0.00 **
**M17**	(E1, SC) + (GTE, −20%) + β-carotene	4.45 ± 0.00
**M18**	(E1, SC) + (GTE, −20%) + NaCl	4.34 ± 0.00
**M(total)**	(E1, SC) + (GTE, −20%) + all minor ingredients	3.58 ± 0.00 **

^1^ SC = standard concentration/commonly applied concentration.

**Table 3:** Determination of *T*_1_/*T*_2_, Fe (mg/kg) and Cu (mg/kg) measured by NMR, TXRF and GFAAS, respectively, in selected margarine samples. NMR data are depicted as single measurements. TXRF and GFAAS data are depicted as mean ± SD (n = 3). Statistically significant differences between the margarine samples regarding their Fe and Cu content are indicated with different lowercase letters a and b (one-way ANOVA, Holm–Sidak, *p* < 0.05).

Margarine	Ingredients	T_1_/T_2_	Fe (mg/kg)	Cu (mg/kg)
**M1**	E1, SC ^1^	2.67	2.88 ± 0.85 ^a^	0.10 ± 0.01 ^a^
**M8**	M1 + aTA, −20%	1.47	3.08 ± 0.56 ^a^	0.06 ± 0.01 ^b^
**M11**	M1 + RE, −20%	3.81	4.64 ± 0.31 ^b^	0.06 ± 0.01 ^ab^
**M14**	M1 + GTE, −20%	33.2	2.22 ± 0.26 ^a^	0.09 ± 0.01 ^ab^
**M(total)**	M14 + minor ingredients	3.58	3.42 ± 0.33 ^ab^	0.06 ± 0.01 ^ab^

^1^ SC=standard concentration/commonly applied concentration.

## Data Availability

Data is contained within the article or supplementary material.

## Supplementary Materials

The following are available online at https://www.mdpi.com/2076-3921/10/1/105/s1, Figure S1: Example of spectral analysis for an inversion recovery experiment. Spectra are shown as dotted lines and fits as blue lines for an inversion recovery experiment. The recovery delay times are indicated above each panel, Figure S2: Example of spectral analysis for a CPMG experiment. Spectra are shown as dotted lines and fits as blue lines for an inversion recovery experiment. The recovery delay times are indicated above each panel.
