# Supplementary material for: Ingredient-Dependent Extent of Lipid Oxidation in Margarine
Source: Antioxidants (Basel). 2021 Jan 13;10(1):105. doi: 10.3390/antiox10010105 (PMC7828556; doi:10.3390/antiox10010105)
Supplement: Supplementary file 1 [file antioxidants-10-00105-s001.pdf]

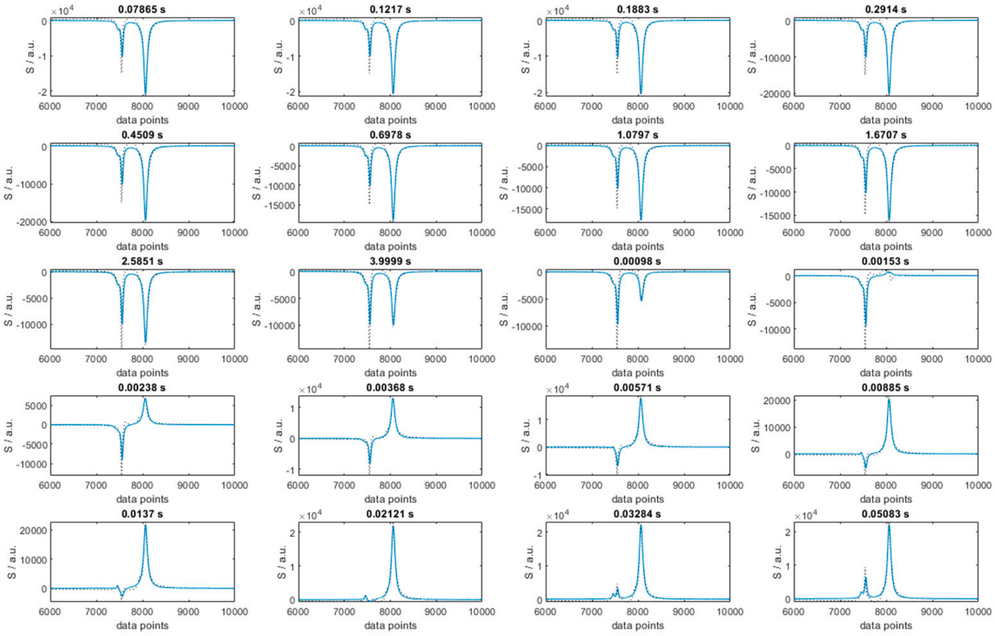

Figure S1. Example of spectral analysis for an inversion recovery experiment. Spectra are shown as dotted lines and fits as blue lines for an inversion recovery experiment. The recovery delay times are indicated above each panel.

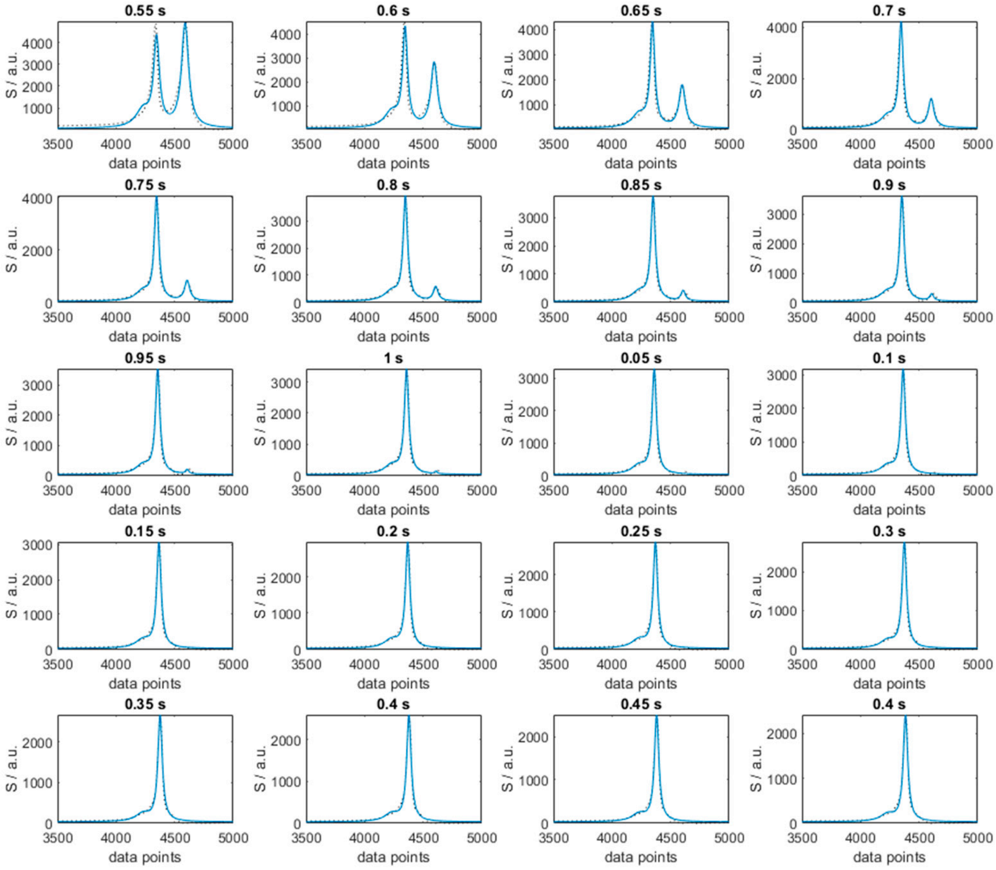

Figure S2. Example of spectral analysis for a CPMG experiment. Spectra are shown as dotted lines and fits as blue lines for an inversion recovery experiment. The recovery delay times are indicated above each panel.
